# Mechanisms of outer membrane vesicle entry into host cells

**DOI:** 10.1111/cmi.12655

**Published:** 2016-09-16

**Authors:** Eloise J. O'Donoghue, Anne Marie Krachler

**Affiliations:** ^1^ University of Birmingham, Institute of Microbiology and Infection, School of Biosciences, EdgbastonBirminghamB15 2TTUK

## Abstract

Bacterial outer membrane vesicles (OMVs) are nano‐sized compartments consisting of a lipid bilayer that encapsulates periplasm‐derived, luminal content. OMVs, which pinch off of Gram‐negative bacteria, are now recognized as a generalized secretion pathway which provides a means to transfer cargo to other bacterial cells as well as eukaryotic cells. Compared with other secretion systems, OMVs can transfer a chemically extremely diverse range of cargo, including small molecules, nucleic acids, proteins, and lipids to proximal cells. Although it is well recognized that OMVs can enter and release cargo inside host cells during infection, the mechanisms of host association and uptake are not well understood. This review highlights existing studies focusing on OMV‐host cell interactions and entry mechanisms, and how these entry routes affect cargo processing within the host. It further compares the wide range of methods currently used to dissect uptake mechanisms, and discusses potential sources of discrepancy regarding the mechanism of OMV uptake across different studies.

## 1 INTRODUCTION

Outer membrane vesicles (OMVs) are nano‐sized proteoliposomes shed from the cell envelope of all Gram negative species of bacteria studied to date (Amano, Takeuchi, & Furuta, 2010). Originally considered an artefact of cell wall turnover or lysis, their release from the bacterial cell is now recognized as a generalized secretion system that contributes to enhanced fitness, and facilitates interactions between cells in the context of mixed bacterial communities and during host–microbe interactions (Bonnington & Kuehn, 2014; Haurat, Elhenawy, & Feldman, 2015).

OMVs are typically 20–200 nm in diameter, and are released during all growth phases and in all environmental conditions studied to date (Bonnington & Kuehn, 2014). OMVs are capable of delivering a chemically diverse range of cargo over long distances while protecting vesicular contents from the external environment (Bonnington & Kuehn, 2014). Cargo can either be contained as a solute within the vesicle lumen, or can be incorporated into or associated with the membrane bilayer, and can include nucleic acids such as siRNA and DNA, toxins, and cell wall components such as peptidoglycan and lipopolysaccharide (LPS; Renelli, Matias, Lo, & Beveridge, 2004; Lindmark et al., 2009; Kaparakis et al., 2010; Vanaja et al., 2016; Koeppen et al., 2016). Due to their versatility as a delivery vehicle, the contributions of OMVs to bacterial fitness are equally varied, but of increasing interest is their role in host colonization and disease pathogenesis (Kuehn & Kesty, 2005). Secretion of OMVs is generally considered an adaptive response to stress, and infection often occurs within a stressful environment (MacDonald & Kuehn, 2012). In some cases, increased production of OMVs under stressful conditions is correlated with increased survival, such as in the presence of antimicrobial peptides, and vesiculation increased resistance to bacteriophage infection (Manning & Kuehn, 2011). Pathogenic species of bacteria generally release more OMVs than their non‐pathogenic counterparts, and it is likely that OMV secretion has been adapted by pathogens to enhance their virulence (Horstman & Kuehn, 2000). *Pseudomonas aeruginosa* OMVs isolated from a cystic fibrosis patient showed a 3–4 fold higher association with lung cells than OMVs from a lab adapted strain, PAO1, via the interaction between vesicle‐associated Pseudomonas aminopeptidase (PaAp) and the lung cells, suggesting an important role for OMV cargo in an infection setting (Bauman & Kuehn, 2009).

OMVs have defensive roles during infection, by sequestering antibiotics and antibodies, as well as acting as decoy antigens to divert the attention of the immune system away from the bacterial cell (Chattopadhyay & Jaganandham, 2015; Ellis & Kuehn, 2010; Vidakovics et al., 2010). The potency of OMVs as offensive weapons is demonstrated by their ability to induce fatal sepsis even in the absence of intact bacterial cells (Park et al., 2010).

OMVs are also able to deliver a selection of virulence factors, including toxins, adhesins, and immunomodulatory molecules directly into host cells during infection (Alaniz, Deatherage, Lara, & Cookson, 2007; Lindmark et al., 2009; Roy, Hamilton, Munson, & Fleckenstein, 2011). While many of these virulence factors have now been verified as OMV cargo, with some even preferentially secreted via this pathway, such as the cytolysin ClyA in enterohemorrhagic E. coli (EHEC), heat labile enterotoxin (LT) in enterotoxigenic E. coli (ETEC), and the vacuolating toxin VacA in *Helicobacter pylori*, identifying the processes which enable the delivery of OMV associated cargo into host cells has proved challenging (Horstman & Kuehn, 2000; Wai et al., 2003; Ricci et al., 2005). This review aims to identify common and contrasting mechanisms which enable OMV entry and cargo delivery to host cells during infection.

**Figure 1:**
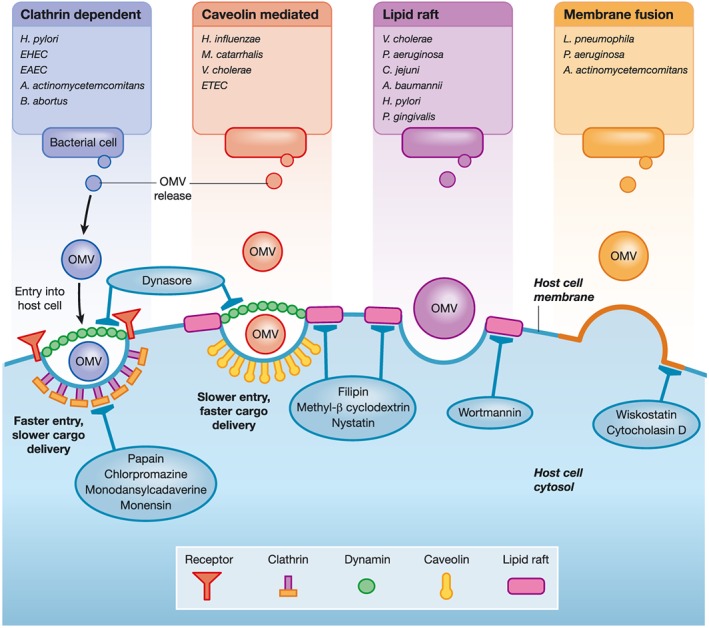
Routes of OMV entry into host cells. Several different pathways allowing OMVs from a variety of Gram negative species of bacteria to enter host cells have been described. These routes can require clathrin coated pits, formation of caveolae, and use of lipid rafts or direct membrane fusion. OMV entry can be impaired by the use of inhibitors against components of these pathways: chlorpromazine–inhibits clathrin coated pit formation; papain–proteolytically degrades surface protein receptors; monensin–ionophore, dissipates proton gradient; monodansylcadaverine–inhibits receptor internalization; dynasore–inhibits dynamin GTPase activity; methyl‐β cyclodextrin–extracts cholesterol from membrane; filipin and nystatin–intercalate and disrupt cholesterol‐rich membrane domains; wortmannin–inhibits phosphatidylinositol kinases; wiskostatin–inhibits N‐WASP, which regulates actin polymerization; cytocholasin D–depolymerises actin

Endocytosis is a process by which small molecules can cross the membrane bilayer of a cell (Doherty & McMahon, 2009). In non‐phagocytic cells, there are four main pathways for the entry of small solutes: Macropinocytosis, clathrin mediated endocytosis, caveolin mediated endocytosis, or non‐caveolin, non clathrin mediated endocytosis (Rewatkar, Parton, Parekh, & Parat, 2015). These pathways have all been implicated in mediating OMV entry into host cells (Figure 1).

## 2 MACROPINOCYTOSIS

Macropinocytosis is characterized by the formation of large (over 200 nm in diameter), actin‐driven, ruffled protrusions from the cell membrane, which allow the sampling and internalization of extracellular medium (Weiner et al., 2016). Its role in infection has been observed for *Shigella flexneri*, which invades host cells via macropinosomes (Weiner et al., 2016). The pathway is also utilized by viruses, which are comparable in size to OMVs (Mercer & Helenius, 2008). It has therefore been suggested that OMVs can enter host cells via macropinocytosis (Kaparakis‐Liaskos & Ferrero, 2015). Inhibition of actin polymerization by cytochalasin D or wiskostatin has been observed to reduce the entry of OMVs from P. aeruginosa into airway epithelial cells (Bomberger et al., 2009). However, macropinocytosis is generally not a cargo induced process, and it is likely that entry via this route is not a deliberate OMV‐driven event (Lim & Gleeson, 2011). Treatment with actin inhibitors is not entirely specific for macropinocytosis; movement of endosomes also requires actin remodeling, and so reduced cargo delivery after these treatments may also be due to the inadvertent effect on other endocytic routes (Soldati & Schliwa, 2006). Macropinocytosis allows internalization of endocytic vesicles up to 1 um in diameter, whereas clathrin dependent and caveolin or lipid raft mediated endocytosis generally allows internalization of considerably smaller cargo (120 nm, 60 nm, and 90 nm respectively; Amano et al., 2010). The size of OMVs ranges from 20 to 500 nm, and this diversity may influence their preferred route of uptake (Amano et al., 2010; Kaparakis‐Liaskos & Ferrero, 2015).

## 3 CLATHRIN DEPENDENT ENDOCYTOSIS

Clathrin mediated endocytosis occurs via the formation of clathrin coated pits up to 200 nm in diameter (Vercauteren et al., 2010). Unlike macropinocytosis, internalization can be triggered by ligand binding to cell surface receptors (Rewatkar et al., 2015). Budding off of the vesicle requires dynamin, and the internalized vesicle enters the endosomal trafficking routes, from where its cargo can be returned to the cell surface or targeted to lysosomes for degradation (Ritter et al., 1995). Many bacterial virulence factors, such as shiga toxin, cholera toxin, and the arg‐gingipain adhesin of *Porphyromonas gingivalis* have been shown to utilize clathrin mediated endocytosis to gain entry into host cells during infection (Boisvert & Duncan, 2008.

Sandvig & van Deurs, 2002). Because OMVs are known to transport various virulence factors during infection, it is reasonable to infer that they can utilize toxin‐receptor interactions to facilitate their cargo delivery via clathrin dependent endocytosis. Clathrin mediated endocytosis is typically inhibited using drugs such as chlorpromazine to prevent formation of clathrin coated pits, or dynamin inhibitors to prevent scission of the endosome from the membrane (Vercauteren et al., 2010).

Several studies have identified clathrin mediated endocytosis as a route for OMV entry. Vacuolating toxin VacA in *H. pylori* is an important cytotoxic virulence factor that is found in OMVs during infection (Parker, Chitcholtan, Hampton, & Keenan, 2010). VacA containing OMVs entered host cells more efficiently than their VacA deficient counterparts, in a cholesterol independent fashion, but inhibition of clathrin mediated endocytosis by chlorpromazine had a stronger inhibition on VacA deficient OMVs, suggesting that VacA is not a receptor ligand but may enable the OMVs to adapt to use alternative pathways in the absence of the clathrin mediated pathway (Parker et al., 2010). The OMVs were labeled with the lipophilic dye DiO, and intracellular fluorescence was measured using flow cytometry (Table 1). It is not clear whether membrane labeling of OMVs affects their function or interaction with the membranes of host cells, and the affinity of lipophilic dyes for plasma membranes necessitates stringent controls and washing steps to ensure the dye does not label the cell membrane in addition to the vesicle (Mulcahy, Pink, & Carter, 2014). Lipophilic dye molecules have been extensively used due to their efficient incorporation into membranes. However, the dye molecules can also form aggregates and enriched domains resulting in changes to the mobility and stiffness of the lipid bilayer, and these physical alterations may in turn affect the behavior of the labeled membrane (Lulevich, Shih, Lo, & Liu, 2009).

**Table 1:** Overview of methods to determine OMV uptake by host cells, their advantages and disadvantages

Method of detecting OMV uptake by host cells	External	Bound	Internal	Advantages of method	Disadvantages	References
Antibody staining	✓***□***	✓***□***	✓***□***	Shows delivery of OMV cargo Allows study of contributions of cargo to interactions with host cell for binding/entry processes or downstream cellular effects Enables visualization of colocalization with particular cellular compartments Detection via flow cytometry or microscopy	May obscure OMV epitopes that facilitate uptake Requires prior knowledge of OMV cargo and so may ignore subpopulations that are not detected with the antibody No data on kinetics of uptake due to requirement of fixation at pre‐determined time points Need high concentrations of OMVs and epitopes in order to visualize with immunofluorescence microscopy	Furuta et al., 2009; Guidi et al., 2013; Jin et al., 2011; Kaparakis et al., 2010; Kim et al., 2010; Kunsmann et al., 2015; Mondal et al., 2016; Parker et al., 2010; Rompikuntal et al., 2012; Thay et al., 2014; Vanaja et al., 2016
Lipophilic dyes for membrane labeling eg. DiO, PKH26	✓***□***	✓***□***	✓***□***	Allows labeling of the whole OMV population Can determine interaction between OMV membrane and host receptors or lipid raft regions Can be used on live cells to resolve kinetics of uptake	Requires controls to prevent labeling of host cell membrane with excess dye Washing steps to remove extracellular vesicles Membrane labeling may affect normal behavior of the OMV and its interactions with the host cell membrane Often required in combination with antibody labeling to prove the labeled membrane is OMV derived	Guidi et al., 2013; Kunsmann et al., 2015; Parker et al., 2010; Thay et al., 2014; Waller et al., 2016
Rhodamine R18		✓***□***	✓***□***	Allows labeling of the whole OMV population Can determine interaction between OMV membrane and host receptors or lipid raft regions Can be used on live cells to resolve kinetics of uptake	Requires controls to prevent labeling of host cell membrane with excess dye Washing steps to remove extracellular vesicles Membrane labeling may affect normal behavior of the OMV and its interactions with the host cell membrane Often required in combination with antibody labeling to prove the labeled membrane is OMV derived	Bomberger et al., 2009; Rompikuntal et al., 2012;
FITC labeling	✓***□***	✓***□***	✓***□***	Allows non‐specific labeling of OMV proteins Can be used in live cells to resolve kinetics Allows detection of OMVs outside and inside host cells	Unknown effects on natural OMV behaviors or uptake processes Non‐specific so often required in combination with antibody labeled components	Chatterjee & Chaudhuri, 2011; Kesty et al., 2004; Pollak et al., 2012; Schaar et al., 2011; Sharpe et al., 2011
OMV targeted GFP	✓***□***	✓***□***	✓***□***	No processing required Can be used in live cells Strongly fluorescent and specific to OMVs No observable effects on OMVs or host cells Adaptable platform for study of OMVs	Targeting sequence specific for E. coli, not tested for other species Need to engineer and verify strain prior to use	Kim et al., 2008.
OMV labeled with cleavable disulphide linker	***✓□***	✓***□***	✓***□***	Distinguishes bound from internalised OMVs Sensitive system with low background Doesn't require prior knowledge of OMV cargo or epitopes Can quantify OMV uptake and is adaptable for high throughput applications	Requires wash steps and controls to ensure reduction of linker Cells require fixation at pre‐determined time points Membrane labeling of OMVs has unknown effects on their behavior.	Olofsson et al., 2014

Contradictory findings to work from Parker et al. were presented by Kaparakis et al. (2010), who observed that entry of *H. pylori* OMVs was dependent on lipid rafts, and entry was significantly reduced after sequestration of cholesterol from the host cell membrane. A similar finding was also observed by Olofsson et al, which demonstrated a role for lipid‐raft associated cholesterol in entry of *H. pylori* OMVs, which was inhibited by treatment with methyl‐b cyclodextrin or filipin (Olofsson et al., 2014). OMV release is a conserved phenomenon, but there are considerable differences in composition and activity of OMVs between species, between strains, and even between the same strains under different external pressures (McBroom & Kuehn, 2007). This may explain some of the discrepancies in the data regarding the uptake routes of OMVs from the same species. The study by Kaparakis et al used Alexa Fluor labeled OMVs, with antibody labeling used to determine internalization and lipid raft stains to observe colocalization. However, using light microscopy to observe OMVs can be problematic due to their small size (often less than ~100 nm) and there is a need for a more reliable and high resolution method of quantifying and identifying internalization of OMVs, particularly when attempting to assess colocalization of OMVs with particular compartments of the cell (Mulcahy et al., 2014). Furthermore, antibody labeling may obscure OMV epitopes important in determining association with host receptors and thus, entry mechanism. Uptake of OMVs has been shown to be a rapid process, with internalization detected as little as 15 min following infection (Wai et al., 2003). Many methods involving use of immunofluorescence microscopy require fixation at pre‐determined time points, and a live cell imaging method would be beneficial to define the kinetics of OMV interactions with host cells.

Methods used to isolate OMVs can also vary, with most using ultracentrifugation but others using sucrose gradients or commercially available isolation columns (Chutkan, Macdonald, Manning, & Kuehn, 2013). The size of the OMV population is relevant when studying endocytic routes; clathrin mediated endocytosis generally allows internalization of larger cargo than clathrin‐independent routes (El‐Sayed & Harashima, 2013). Different isolation methods can introduce a bias towards particular sizes of OMVs, for example with the use of filters to exclude particles over 200 nm in diameter, and the lack of standardized isolation procedures may also explain some of the differences in findings in studies of OMVs from the same species(Kulp & Kuehn, 2010).

Other evidence for the entry of OMVs via receptor mediated endocytosis was recently described by Vanaja et al. (2016) who showed that in cells with an siRNA knockdown of AP2, an adaptor protein required for internalization of clathrin coated pits, there was a reduced response to the LPS delivered by EHEC OMVs. This indicated a reduction in the ability of the OMVs to enter the cell, which was also observed when the LPS of the OMVs was neutralized with polymyxin B, suggesting a functional link between LPS, clathrin and the induction of inflammatory responses (Vanaja et al., 2016). The fate of the LPS was to escape the endosomal compartments and induce caspase‐11 activity, causing cytokine production and cell death. LPS is a highly immunogenic component of OMVs,(Vanaja et al., 2016) and modification of LPS has been used as a way to reduce immunogenicity and enhance the suitability of OMVs as an adjuvant in vaccine preparations (Kim et al., 2009). The role of LPS during OMV host cell interactions is thus an attractive and important area for further investigation.

Caspase induction was also observed after incubation with enteroaggregative E. coli O104:H4 OMVs (Kunsmann et al., 2015). Labeled OMVs were found to contain several antigens, including shiga toxin, flagellin and enterotoxin, and caused cell death by the induction of caspase‐9 mediated apoptosis, and inflammation through increased IL‐8 release (Kunsmann et al., 2015). Treatment with dynasore and chlorpromazine significantly reduced the uptake of OMVs, suggesting entry of OMVs and their cargo occurs via the receptor mediated endocytic pathway (Kunsmann et al., 2015). Neutralization of OMV LPS with polymyxin B reduced the secretion of IL‐8, in agreement with other studies indicating a role of LPS in driving pro‐inflammatory responses (Kunsmann et al., 2015; Vanaja et al., 2016).

OMVs from EHEC containing the hemolysin HlyA were shown to enter host cells, with HlyA released from lysosomes into the cytoplasm where it was then trafficked to mitochondria, resulting in caspase‐3 and caspase‐9 activation and subsequent death of epithelial and endothelial cells (Bielaszewska et al., 2013). Treatment with dynasore and chlorpromazine significantly reduced OMV entry, suggesting EHEC‐HlyA OMVs enter via clathrin mediated endocytosis. Fluorescence microscopy confirmed the colocalization of HlyA and clathrin, while there was no colocalization observed between HlyA and caveolin (Bielaszewska et al., 2013). When free HlyA was added to the cells, it remained at the cell surface and was not internalized, suggesting that the association with OMVs is necessary to allow efficient delivery into the cell (Bielaszewska et al., 2013).

Many studies have demonstrated a role for clathrin in the internalization of OMVs, but with the caveat that OMVs are able to compensate well in the absence of this entry route. Similarly to the finding by Parker et al that OMVs can utilize more than one route of entry, OMVs from *A. actinomycetemcomitans* showed a 25% reduction in uptake when clathrin mediated endocytosis was inhibited by monensin, and an equivalent reduction when cholesterol was bound by filipin (Parker et al., 2010; Thay et al., 2014). OMVs from *Brucella abortus* were also shown to enter monocytes primarily via a clathrin dependent route, with monodansylcadaverine treatment resulting in a 33% inhibition of OMV entry, and no effect seen after filipin treatment (Pollak, Delpino, Fossati, & Baldi, 2012). However, the partial level of inhibition implies that the OMVs are able to use alternative pathways. Interestingly, the study also showed that pre‐incubation with OMVs prior to infection with whole cells inhibited the TNF‐⟨ responses, and increased the numbers of internalized *B. abortus*, demonstrating a role for OMVs in immunomodulation during or prior to subsequent infection. The ability of toll‐like receptors to activate upon addition of their agonists was also reduced after pre‐treatment with OMVs (Pollak et al., 2012). This study was conducted with monocytes rather than epithelial cells and there may be differences in entry of OMVs into phagocytic cells compared with non‐phagocytic cell lines used in many studies (Pollak et al., 2012).

Incomplete levels of inhibition were also seen in studies with *H. pylori* OMVs, with a method termed ‘Quantification of internalised substances’ which labeled the *H. pylori* OMVs with a dye containing a cleavable disulphide bond, allowing quenching of extracellular OMV‐associated fluorescence by the addition of a reducing agent (Olofsson et al., 2014; Table 1). Fluorescence inside the epithelial cells was then assessed with microscopy (Olofsson et al., 2014). This work demonstrated involvement of dynamin, with dynamin inhibition causing an 80% reduction in internalization, but chlorpromazine only reducing internalization by 40% (Olofsson et al., 2014). Dynamin is involved in both clathrin mediated and caveolin mediated endocytosis, and so it appears that there is a contribution of both clathrin mediated and caveolin mediated endocytosis towards OMV entry (Vercauteren et al., 2010).

Entry into a cell via the clathrin mediated endocytic pathway typically utilizes receptor‐ligand binding to drive internalization (El‐Sayed & Harashima, 2013). While this route has been implicated in many studies of OMV entry, the possible ligands have remained elusive. If internalization of OMVs requires these interactions, then identifying the components involved could allow the design of inhibitors to attenuate infections by preventing the delivery of OMV‐associated virulence factors.

## 4 NON CLATHRIN MEDIATED ENDOCYTOSIS

Many studies have indicated a role for lipid rafts in enabling OMV entry(Furuta et al., 2009; Kaparakis et al., 2010; Jin et al., 2011; Schaar et al., 2011; Sharpe, Kuehn, & Mason, 2011; Elmi et al., 2012; Kim et al., 2010; Thay et al., 2014; Mondal et al., 2016) . Lipid rafts are domains of the plasma membrane that are enriched in sphingolipids and cholesterol (Mulcahy et al., 2014). The lipid composition of these domains causes them to be more ordered and compact than neighboring regions (Simons & Ehehalt, 2002). Cholesterol‐rich regions are abundant in the bilayer, and it is hypothesized that clustering of the regions allows curvature of the membrane, driving formation of invaginations in the host cell and entry of particles into the cell (Pelkmans, 2005). It is well‐established that viruses exploit lipid rafts to enter host cells and the similarities between enveloped viruses and OMVs in terms of size and composition would suggest a potential affinity for this route of entry (Kulp & Kuehn, 2010).

Cholesterol is a principal component of lipid raft domains, and cholesterol dependency has been demonstrated for entry of OMVs from a variety of species (Bomberger et al., 2009; Furuta et al., 2009; Kim et al., 2010; Jin et al., 2011; Schaar et al., 2011; Sharpe et al., 2011; Elmi et al., 2012; Olofsson et al., 2014; Thay et al., 2014; Mondal et al., 2016). Cholesterol‐rich microdomains are commonly disrupted by using chemicals such as methyl‐β‐cyclodextrin (mbcd, sequesters and depletes cholesterol from the cell membrane) or filipin (binds to cholesterol in the membrane and disrupts lipid packing, Danthi & Chow, 2004; Vercauteren et al., 2010). Many reports have used this approach to demonstrate the importance of membrane cholesterol for delivery of OMV cargo. OMVs from *Vibrio vulnificus* delivered cytolysins into epithelial cells to induce cell death, but this effect was diminished in the presence of filipin (Kim et al., 2010). Treatment with mbcd prevented delivery of OmpA from *A. baumannii* OMVs to host cells (Jin et al., 2011). OMVs commonly cause immune activation via the induction of cytokines, and their production is measured using ELISAs to determine the level of inflammatory stimulation (Schaar et al., 2011; Sharpe et al., 2011; Elmi et al., 2012; Pollak et al., 2012; Kunsmann et al., 2015; Mondal et al., 2016; Waller et al., 2016). Treatment of host cells with mbcd prior to infection with OMVs from *Campylobacter jejuni* resulted in reduced production of IL‐8, IL‐6 and TNF‐α (Elmi et al., 2012). The cargo of OMVs can also assist in allowing lipid‐raft mediated entry processes. OMVs from a clinical isolate of P. aeruginosa displayed PaAP aminopeptidase on the surface and showed a 40% higher association with lung cells than the OMVs from a PaAP deletion strain, and this association was dependent on membrane cholesterol (Bauman & Kuehn, 2009).

### 4.1 Caveolin mediated endocytosis

Lipid raft domains can also be enriched in caveolin, and the oligomerization of caveolin allows formation of caveolae (Rewatkar et al., 2015). Caveolae are cave‐shaped invaginations that are formed on the cell membrane, around 80 nm in diameter, and enriched in cholesterol, caveolins, and sphingolipids (Mulcahy et al., 2014). Similarly to clathrin mediated endocytosis, dynamin is also required for scission and internalization of caveolae (Rewatkar et al., 2015). Although the speed of caveolae internalization is around five times slower than that of clathrin mediated endocytosis, the efficiency of cargo delivery into the cytosol is much higher (Ritter et al., 1995).

Interactions between pathogens and caveolae have been reported, has and caveolae have been suggested as a preferential invasion mechanism for many pathogens, including bacteria, viruses and fungi, as the internalised caveolae are thought to avoid fusion with lysosomal compartments and subsequent degradation, in contrast to clathrin coated pits (Anderson, Chen, & Norkin, 1996; Long et al., 2012; Lim et al., 2014). E. coli and *Leishmania chagasi* internalized via caveolae are able to persist within macrophages (Baorto et al., 1997; Rodriguez, Gaur, & Wilson, 2006). Chlamydial species are able to avoid detection during intracellular infection by using caveolins to disguise the internalized phagosome as a host‐derived vesicle (Norkin, Wolfrom, & Stuart, 2001). Simian virus 40 (SV40) also enters host cells through caveolae, and uptake of exosomes produced from cells infected with Epstein Barr virus also requires caveolae (Anderson et al., 1996; Nanbo, Kawanishi, Yoshida, & Yoshiyama, 2013).

There are now numerous examples of OMVs utilizing caveolin mediated endocytosis to enter host cells. However, many studies often fail to distinguish between lipid raft dependency, which is inhibited by cholesterol depletion, and caveolin‐specific lipid raft dependency, which is sensitive to both cholesterol and dynamin depletion. OMVs from non‐typeable *Haemophilus influenzae* were shown to enter and colocalize with caveolin 1 (Cav‐1), a marker of caveolae, by western blotting of epithelial cell lysates after infection (Sharpe et al., 2011). Treatment of cells with filipin to disrupt cholesterol rich microdomains in the membrane abolished this interaction. The same study showed that while binding of OMVs to the cell membrane could occur at 4°C, internalization only occurred after incubation at 37°C. This is in agreement with work by Kesty et al. (2004) which showed reduced entry of ETEC OMVs into HT29 intestinal epithelial cells at 4°C compared with 37°C, and also by Jager et al. (2014) which demonstrated the temperature dependence of uptake for OMVs from *Legionella pneumophila*, suggesting that OMV entry is not a passive process.

Caveolin mediated endocytosis of OMVs has been found in many cases to utilize interactions between bacterial ligands and host cell receptors. OMVs from *Moraxella catarrhalis* entered human epithelial cells via interactions between toll‐like receptor 2 (TLR2) and lipid rafts (Schaar et al., 2011). Internalization of FITC‐labeled OMVs was not observed after treatment with filipin, suggesting that the receptors were localized in cholesterol‐rich regions of the membrane. Cholera toxin (CTx) is a virulence factor of *Vibrio cholerae* known to bind to the ganglioside GM1 present in caveolin enriched lipid rafts on the host cell surface, and is secreted in both soluble and OMV‐associated forms (Chatterjee & Chaudhuri, 2011). During infection of intestinal epithelial cells, OMV‐associated CTx was shown to rapidly target GM1 after only 15 min and facilitate internalization of the OMVs (Chatterjee & Chaudhuri, 2011). Similarly, entry of ETEC OMVs relied on the association of heat LT contained within the OMV membrane with the toxin receptor, and immunofluorescence microscopy revealed colocalization of labeled caveolin and vesicles (Kesty et al., 2004). OMVs derived from an LT deficient strain showed a 60% lower association with host cells, demonstrating the role of specific OMV cargo in driving uptake processes. Together, these reports suggest that OMVs from pathogens are adapted for delivery of virulence factors (Kesty et al., 2004).

### 4.2 Non caveolin mediated endocytosis

Alternatively, lipid raft mediated endocytosis can be independent of caveolin and dynamin and instead require small GTPases (Rewatkar et al., 2015). These GTPase dependent processes are the least well characterized of endocytic routes, but are generally defined as uptake into the cell via uncoated membrane invaginations (Mayor, Parton, & Donaldson, 2014).

OMV‐associated proteases from *V. cholerae* reportedly were delivered into intestinal epithelial cells via this dynamin independent, lipid raft mediated endocytic route (Mondal et al., 2016). Induction of pro‐inflammatory cytokines following infection with OMVs was measured using ELISA, and cytotoxicity determined using flow cytometry. Both responses were reduced after treatment with mbcd to deplete cholesterol, but no effect was observed after dynamin inhibition (Mondal et al., 2016). The oral pathogen *Porphyromonas gingivalis* secretes OMVs containing virulence factors such as gingipains and fimbriae, and these OMVs were shown to enter HeLa and gingival epithelial cells in a Rac1/lipid raft dependent manner, and independent of caveolin, clathrin, and dynamin (Furuta et al., 2009). Interestingly, these OMVs were rapidly directed to lysosomes, but avoided degradation for over 24 hr following entry (Furuta et al., 2009). Despite being unable to deliver their contents into the cytosol, the strong and prolonged acidification of lysosomes induced by the OMVs caused cellular damage, even without delivery of specific virulence factors (Furuta et al., 2009).

OMVs can also influence host cellular responses without entering cells. When all endocytic routes were inhibited, OMVs from *P. gingivalis* were still able to cause suppression of immune signaling and increase tolerance to subsequent infection through the induction of TLR4 on the cell surface of monocytes (Waller et al., 2016). In some cases, OMVs can even cause effects in host cells distal from the initial site of infection and OMV production. OMVs from *Salmonella enterica* were produced by whole bacteria in the *Salmonella* containing vacuole (SCV) and the vesicles were able to escape not just the SCV but the infected host cell, and enter uninfected neighboring cells to deliver the genotoxin cytolethal distending toxin (CDT), revealing the ability of OMVs to migrate from the cell of origin (Guidi et al., 2013).

## 5 MEMBRANE FUSION

Despite the different architecture of the membrane bilayer present in OMVs and that of host eukaryotic cells, membrane fusion has been described as a mechanism for OMV entry into host cells. The self‐quenching fluorescent dye Rhodamine‐R18 was used to label OMVs from P. aeruginosa (Bomberger et al., 2009)*.* When added to host epithelial cells, an increase in fluorescence was observed due to lipid mixing between vesicle and host membrane bilayer, leading to dilution and de‐quenching of the dye. The increase in fluorescence was used as a quantitative determinant of membrane fusion between the OMV and the cell membrane (Bomberger et al., 2009). Lipid rafts were labeled with CTxB subunit, and there was colocalization between sites of membrane fusion and the labeled lipid rafts, and the fusion events were inhibited in the presence of filipin. This indicated that membrane fusion events preferentially occur at lipid raft domains (Bomberger et al., 2009). A similar technique was used to assess membrane fusion between OMVs from *A. actinomycetemcomitans* and HeLa cells, which utilized confocal microscopy to identify colocalization between the toxin component cytolethal distending toxin (CDT) and the labeled lipid rafts and sites of dequenched membrane labeling (Rompikuntal et al., 2012). A caveat of studies employing mbcd and filipin to deduce the involvement of lipid rafts in OMV uptake is that both agents, by disrupting a major constituent of the membrane, affect membrane organization on a large scale and may have effects on processes not limited to lipid rafts.

Model membranes have been utilized to confirm that membrane fusion can occur between OMVs and host cell membranes, despite their structural differences. Phospholipid liposomes mimicking host cell membranes were labeled with a pair of FRET dyes to demonstrate that OMV membrane material from L. monocytogenes can be included into the model host bilayer, by monitoring the change in FRET signal, which increased upon incorporation of OMV membrane due to an increase in surface area (Jager et al., 2014). The fusion events occurred just seconds after addition of OMVs, highlighting the rapid and efficient kinetics behind OMV interactions with host cells (Jager et al., 2014). The interaction was also observed to be partially temperature dependent, with a lower level of incorporation when the experiment was conducted at 4°C compared to 37°C, but there was still a notable level of membrane fusion detected, suggesting that fusion does not entirely depend on active, energetic processes (Jager et al., 2014).

## 6 CONCLUSIONS

There are considerable discrepancies between findings determining OMV entry routes into host cells. Differences in uptake routes between OMVs from different species may well be explained by the fact that OMV composition is adapted to direct vesicles towards a specific uptake route, and thus allow them to undergo ideal processing within the host cell to facilitate infection. However, discrepancies also exist between studies analyzing entry of OMVs from the same species. This may be due to discrepancies in methodologies, such as the isolation and quantification of OMVs, the labeling or imaging techniques, or the strains, host cell types and cell lines used. It is also apparent that OMVs can use multiple routes to enter host cells. As described above, different isolation techniques result in different levels of purity and/or size distributions of the OMV preparations. Quantification of OMVs used for uptake assays also varies between studies, and particularly the use of total protein contained within the OMV preparation as a means of normalization is problematic, as protein content and composition can vary widely between OMV preparations derived under different growth conditions (McBroom & Kuehn, 2007). Despite these challenges, the existing literature clearly demonstrates that OMVs are well adapted to direct and deliver their cargo into host cells. In order to fully elucidate the mechanisms underpinning these processes, it will be necessary to develop a consistent, quantifiable and dynamic approach to measure OMV association, entry and cargo delivery to host cells.

Deepening our understanding of how specific cargo molecules direct OMVs towards a specific uptake route, and thus, determine the fate of vesicular contents within the host cell is essential and will allow the future exploitation of OMVs for medical applications (Berleman & Auer, 2013). OMVs engineered to display a ClyA‐HER2 targeting probe were able to target cells over‐expressing HER2, a common biomarker of cancer cells, and induce cell death and tumor shrinkage via the delivery of siRNA targeting the expression of kinesin spindle protein (Gujrati et al., 2014). Insights on OMV adhesion and entry would allow production of engineered OMVs with high affinity for specific cell types or tissue locations, and enhance their potential as novel therapeutic agents (Alves, Turner, Medintz, & Walper, 2015; Gao et al., 2015).

OMVs have already been incorporated in vaccine preparations, due to their immunogenicity and the display of antigens, which unlike purified antigen or heat‐killed bacteria, closely reflects the native conformation in the bacterial cells of origin. However, fewer risks are associated with the use of OMVs compared to live‐cell vaccine preparations, as OMVs are metabolically inert (Collins, 2011; Acevedo et al., 2014; Brudal et al., 2015). Novel targets against bacterial infection are urgently required due to the increasing prevalence of antibiotic resistance (Kulkarni, Nagaraj, & Jagannadham, 2015). The importance of OMVs in infection is now well demonstrated, with the delivery of active virulence factors and immunomodulatory molecules, as well as their defensive roles, all serving to enhance pathogenesis (MacDonald & Kuehn, 2012). Insights into the mechanisms of OMV entry may enable the design of inhibitors to prevent the delivery of their toxic cargo and attenuate infections without selecting for antibiotic resistance (Kulkarni et al., 2015).
